# Supplementary material for: Ageing-Related Changes to H3K4me3, H3K27ac, and H3K27me3 in Purified Mouse Neurons
Source: Cells. 2024 Aug 21;13(16):1393. doi: 10.3390/cells13161393 (PMC11353134; doi:10.3390/cells13161393)
Supplement: Supplementary file 1 [file cells-13-01393-s001.zip › Supplementary_data/Supplementary_Figures.pdf]

Supplementary Figures

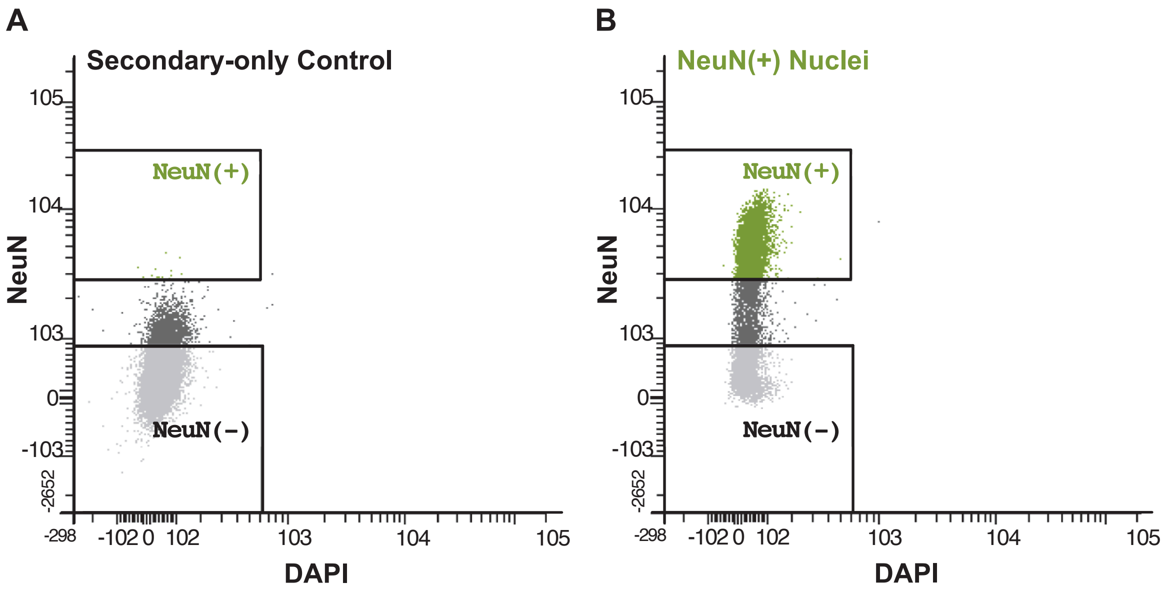

Figure S1. Gating strategy for secondary only control (A) and for purifying NeuN+ (B) mouse forebrain nuclei.

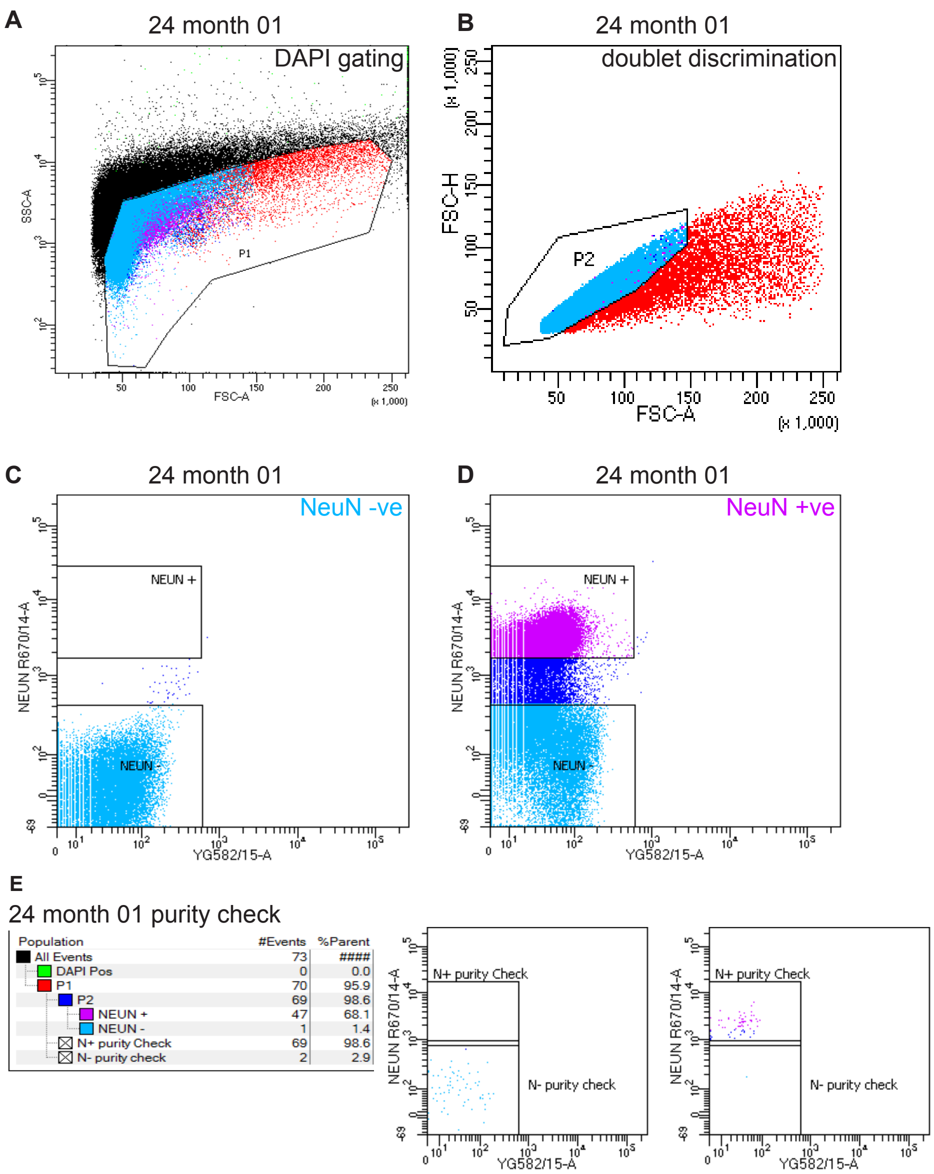

Figure S2. Gating strategy for isolating neuronal nuclei. 4′,6-diamidino-2-phenylindole (DAPI) was used to gate nuclei from cellular debris (A). Single nuclei were isolated via doublet discrimination from the DAPI positive population (B). NeuN negative (C) and positive controls (D) were used to then gate neuronal nuclei, before performing the sort. Sample purity checks were performed to confirm gating strategy for nuclei (E).

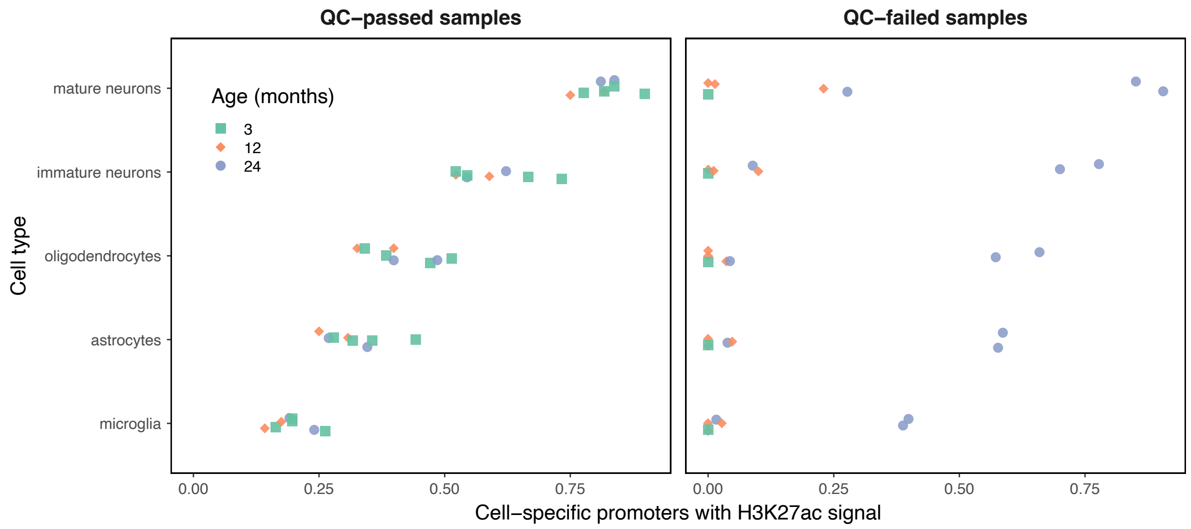

Figure S3. H3K27ac sample coverage of cellular identity gene promoters. To be considered as covering the promoter of a gene, a minimum RPKM of 2 in a 1kb window overlapping the region +/- 2kb from the transcription start site of any protein coding isoforms of the parent gene with a GencodeVM23 transcript support level of 1 or 2 was required. Although not a requirement, all H3K27ac samples which failed QC had a mature neuron signal lower than 0.75, except the 24 month 02 sample which had a low fragment length, and the 24 month 03 sample which had insufficient read depth (Table S1).

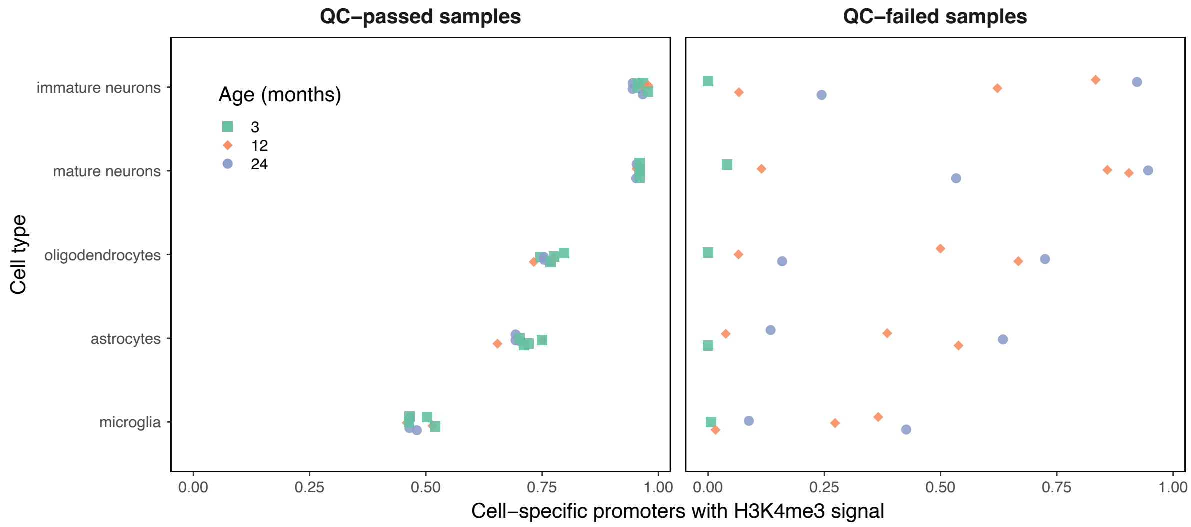

Figure S4. H3K4me3 sample coverage of cellular identity gene promoters. To be considered as covering the promoter of a gene, a minimum RPKM of 2 in a 1kb window overlapping the region +/- 2kb from the transcription start site of any protein coding isoforms of the parent gene with a GencodevM23 transcript support level of 1 or 2 was required. H3K4me3 samples which failed QC all had a mature neuron signal lower than 0.95, and/or failed based on other metrics (Table S1).

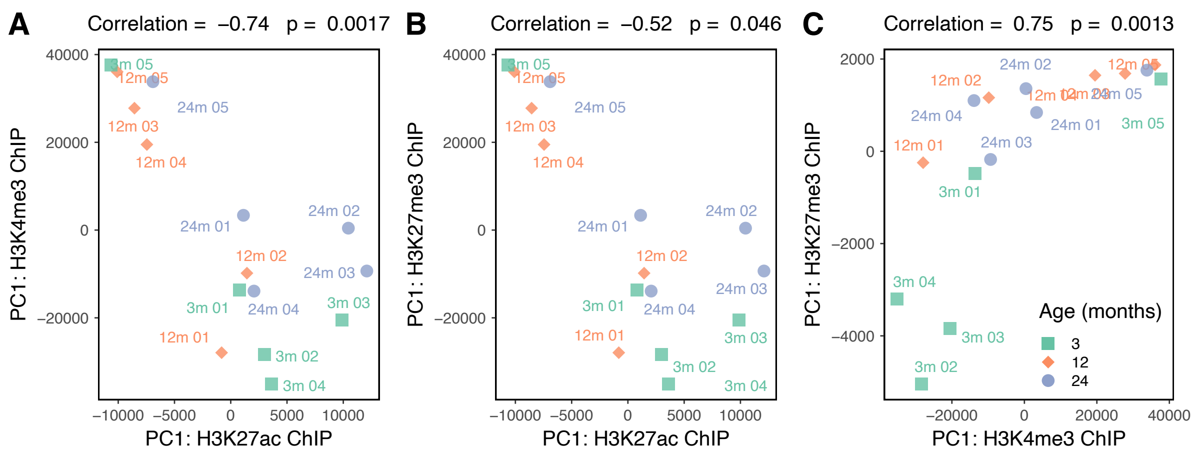

Figure S5. PCA first component correlations across ChIP-Seq marks for all samples. Values for the first principal component of ChIP-Seq signals were plotted against each other, showing a general correlation across histone marks profiled.

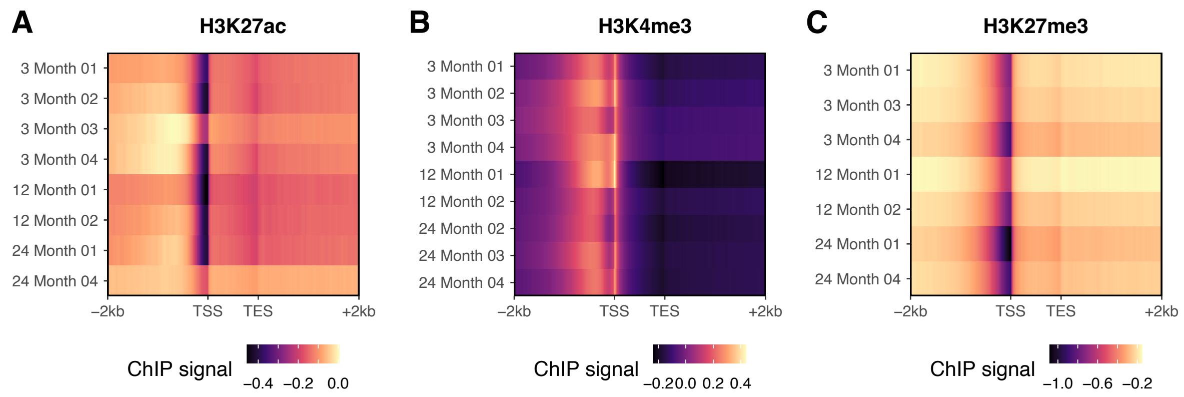

Figure S6. Heatmap profiles of ChIP-Seq signal across Gencode genes in (A) H3K27ac, (B) H3K4me3, and (C) H3K27me3.

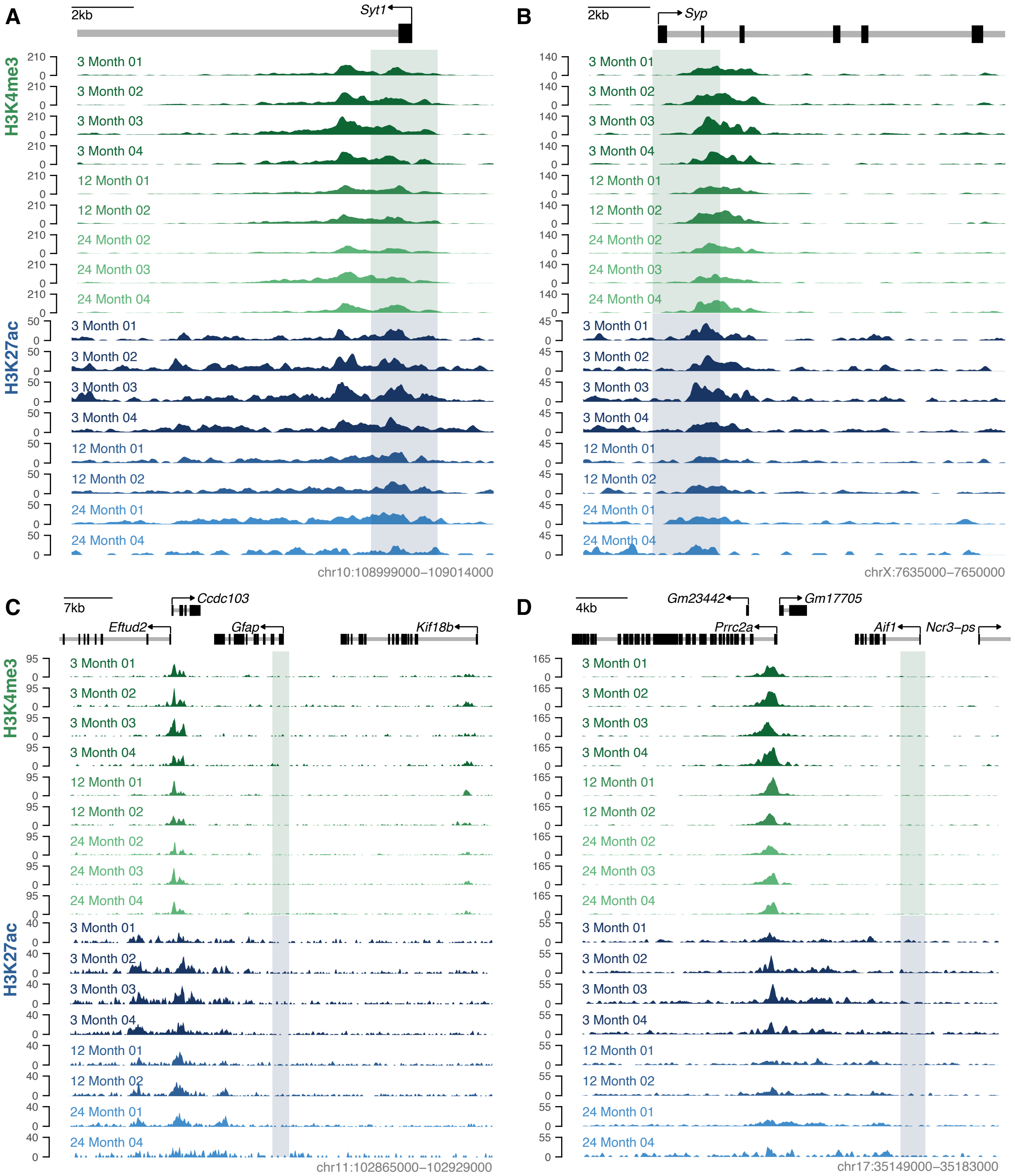

Figure S7. H3K4me3 and H3K27ac neuronal ChIP-seq signal at the neuron-specific genes (A) *Syt1* and (B) *Syp* and glial cell-specific genes (C) *Gfap* and (D) *Aif1.*

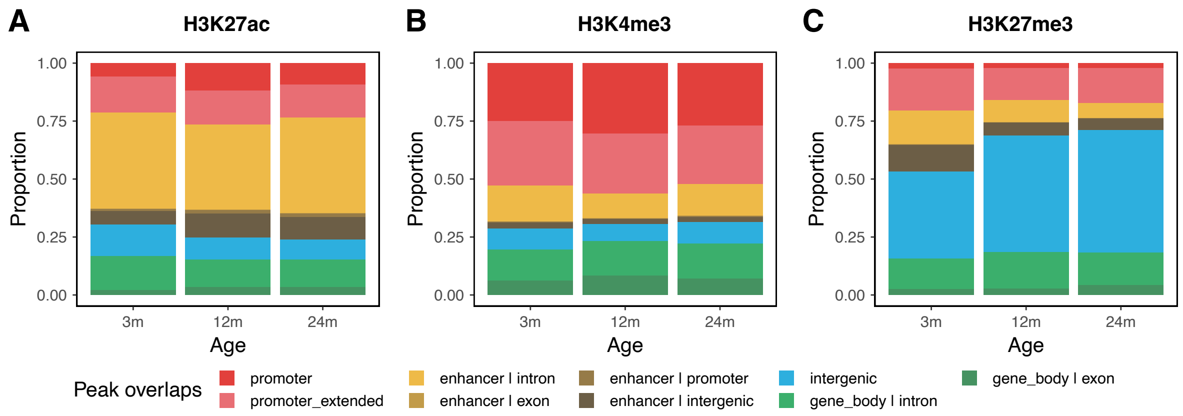

Figure S8. Genomic location and closest annotation of peaks for (A) H3K27ac, (B) H3K4me3, and (C) H3K27me3 at each age.

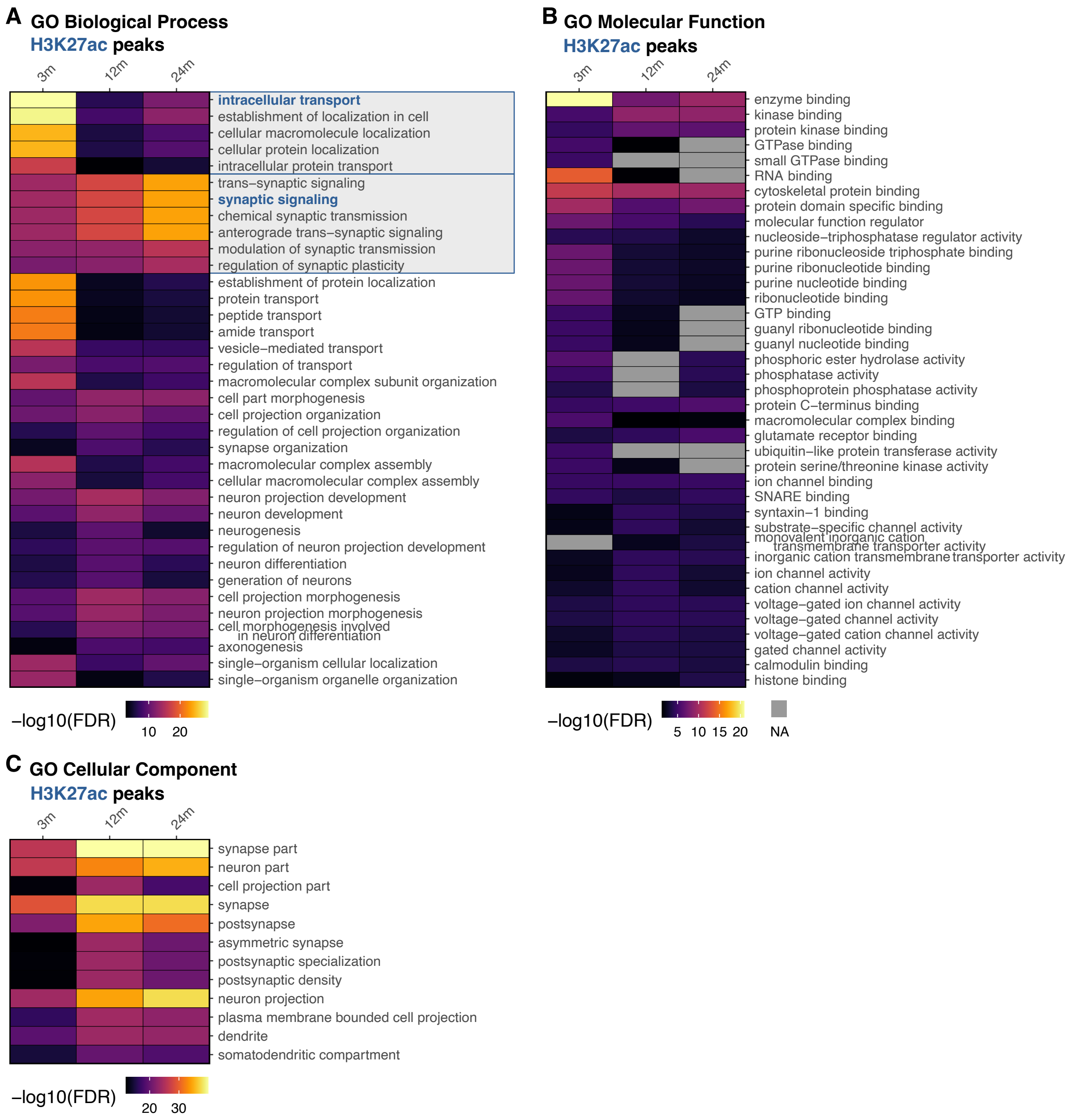

Figure S9. Gene Ontology enrichment for all H3K27ac peaks across ages. (A) Top 20 Biological Process terms by FDR. (B) Top 20 Molecular Function terms by FDR. (C) Top 10 Cellular Component terms by FDR.

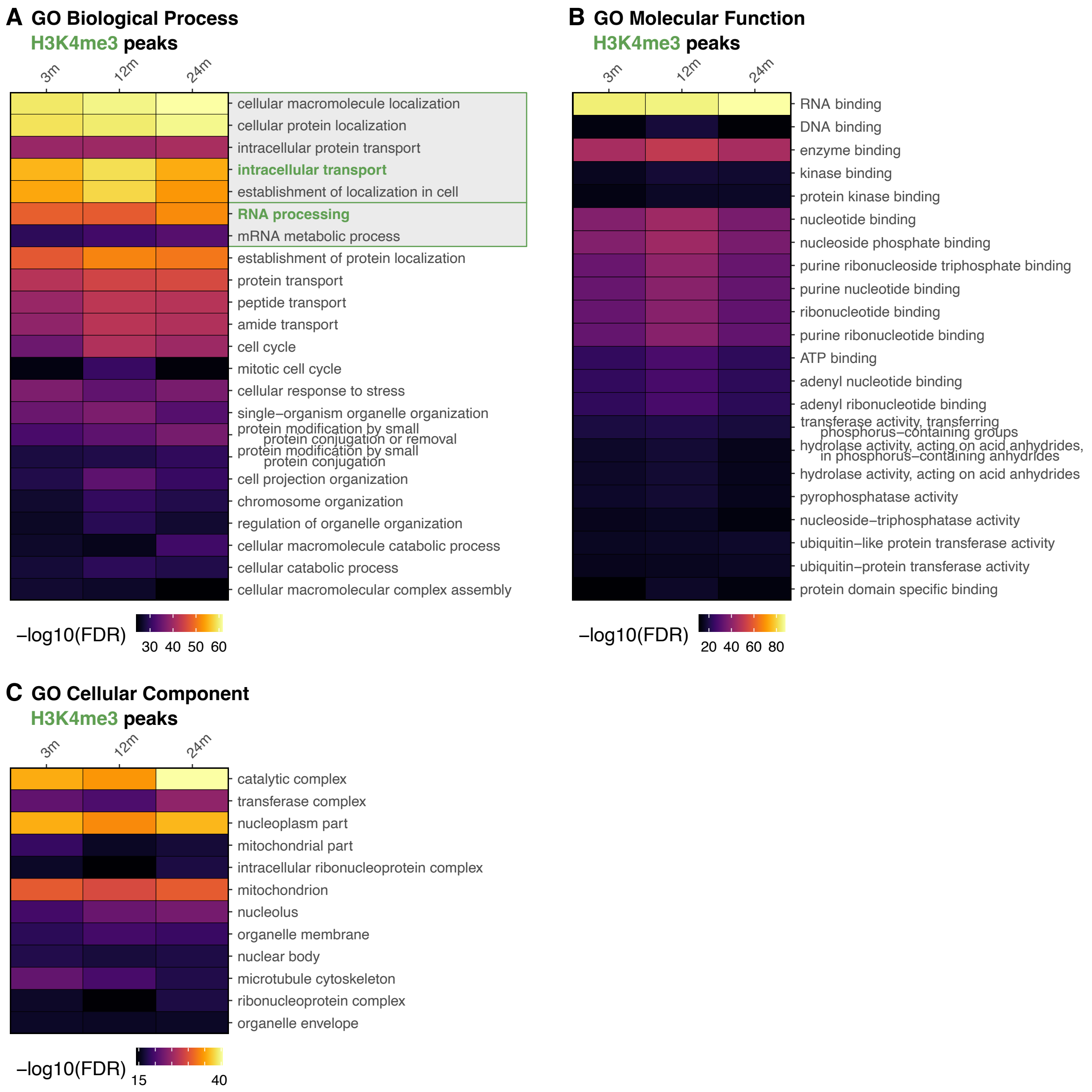

Figure S10. Gene Ontology enrichment for all H3K4me3 peaks across ages. (A) Top 20 Biological Process terms by FDR. (B) Top 20 Molecular Function terms by FDR. (C) Top 10 Cellular Component terms by FDR.

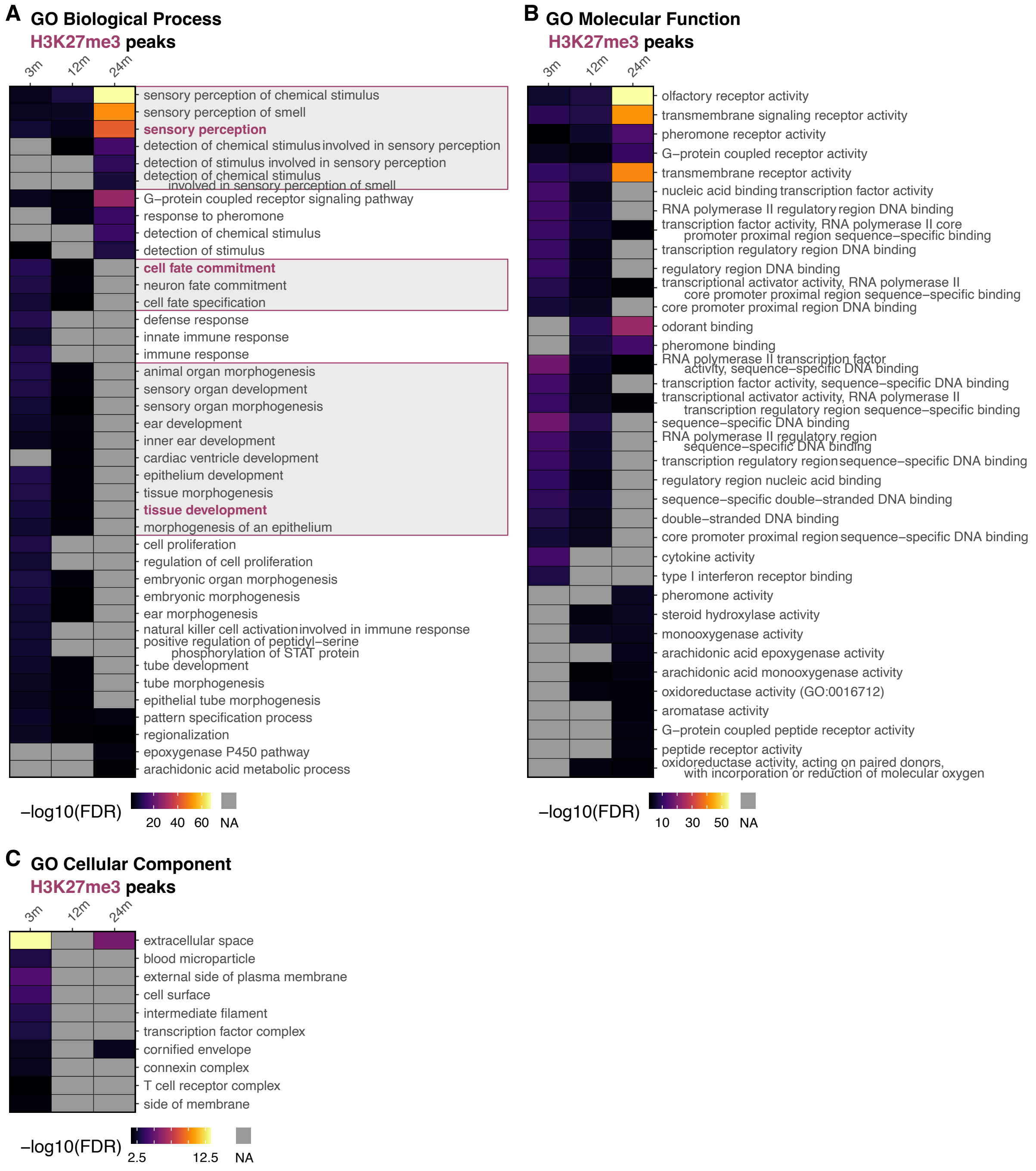

Figure S11. Gene Ontology enrichment for all H3K27me3 peaks across ages. (A) Top 20 Biological Process terms by FDR. (B) Top 20 Molecular Function terms by FDR. (C) Top 10 Cellular Component terms by FDR.

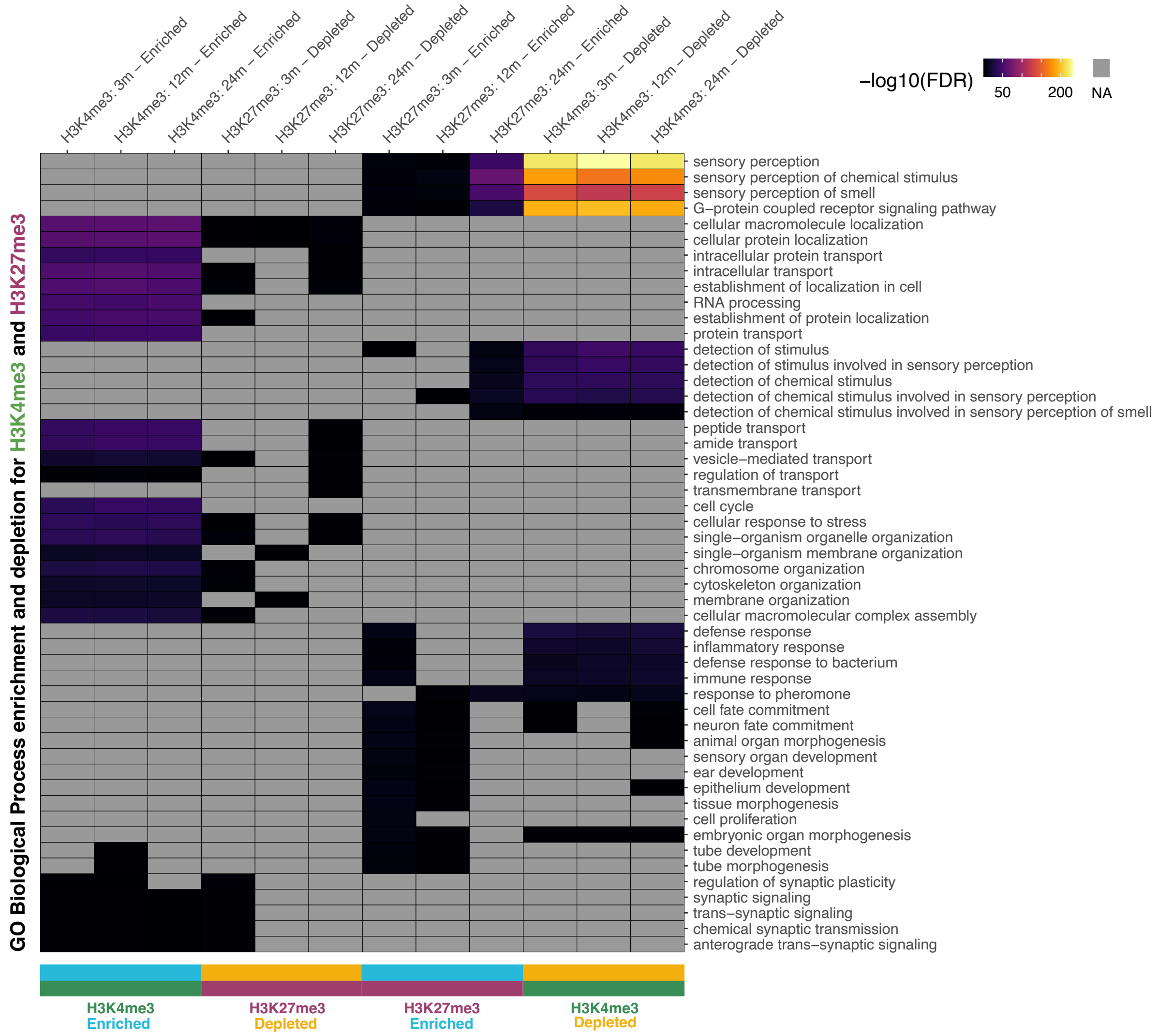

Figure S12. Gene Ontology Biological Process terms enriched and depleted in H3K4me3 mirror terms depleted and enriched in H3K27me3.

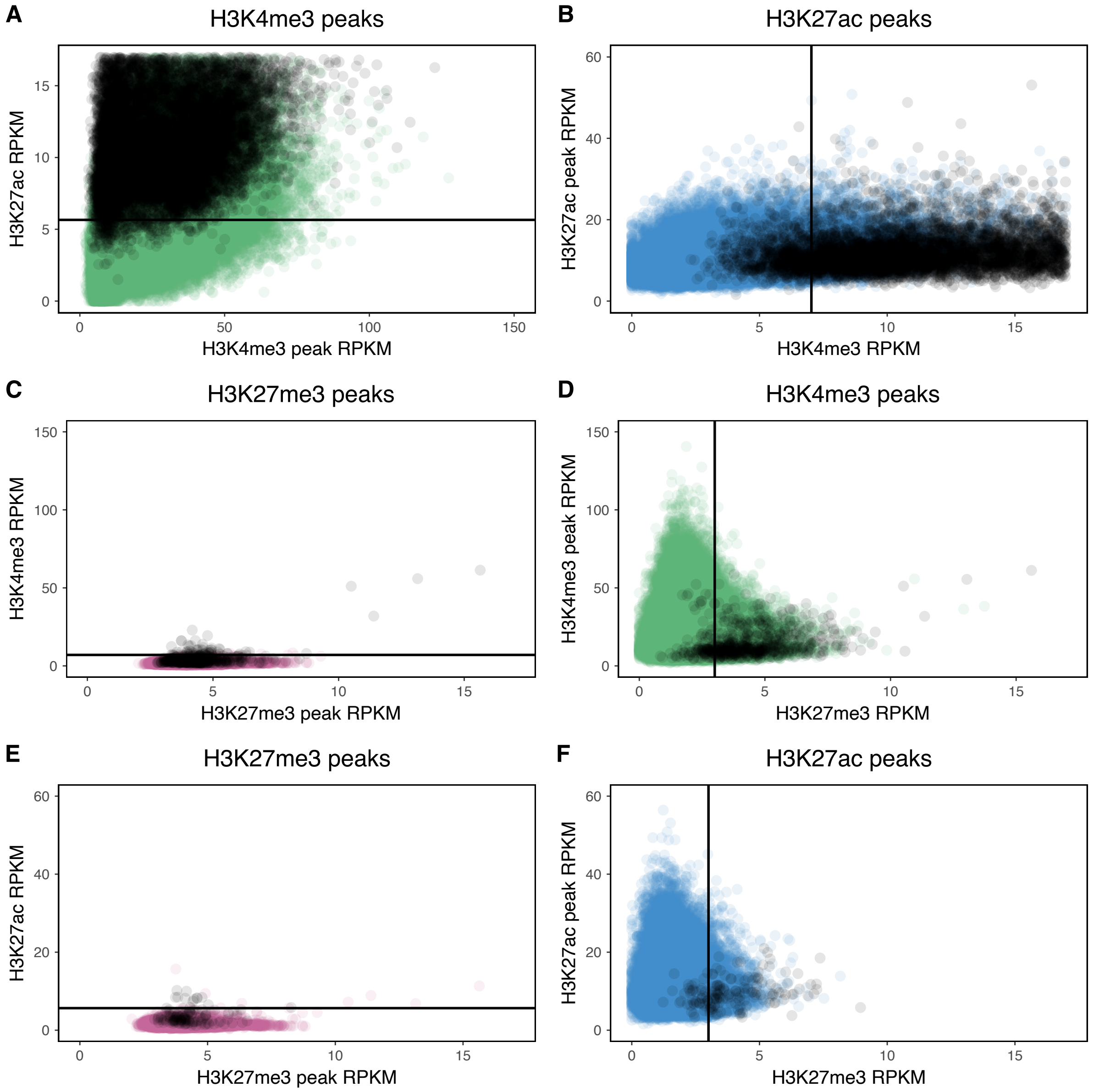

Figure S13. Pairwise overlap between H3K27ac, H3K4me3, and H3K27me3 peak sets. (A-B) comparison of H3K4me3 (green) and H3K27ac (blue) peak sets. (C-D) comparison of H3K27me3 (pink) and H3K4me3 (green) peak sets. (E-F) comparison of H3K27me3 (pink) and H3K27ac (blue) peak sets. For each comparison, one set (indicated by point colour) of peaks was compared to a second set (black). RPKM values shown are the average RPKM values within the peaks, as well as the corresponding, age-matched, RPKM values within the same region from the compared histone modification ChIP-Seq data. Peaks with an overlapping pair in the second set are shown in black, with the proportion of overlap listed in Table S10. The 5th percentile RPKM value (i.e., lower bound) of the full second set, including non-overlapping peaks, is indicated by a horizonal or vertical line. The proportion of peaks that exceed this value in Table S10.

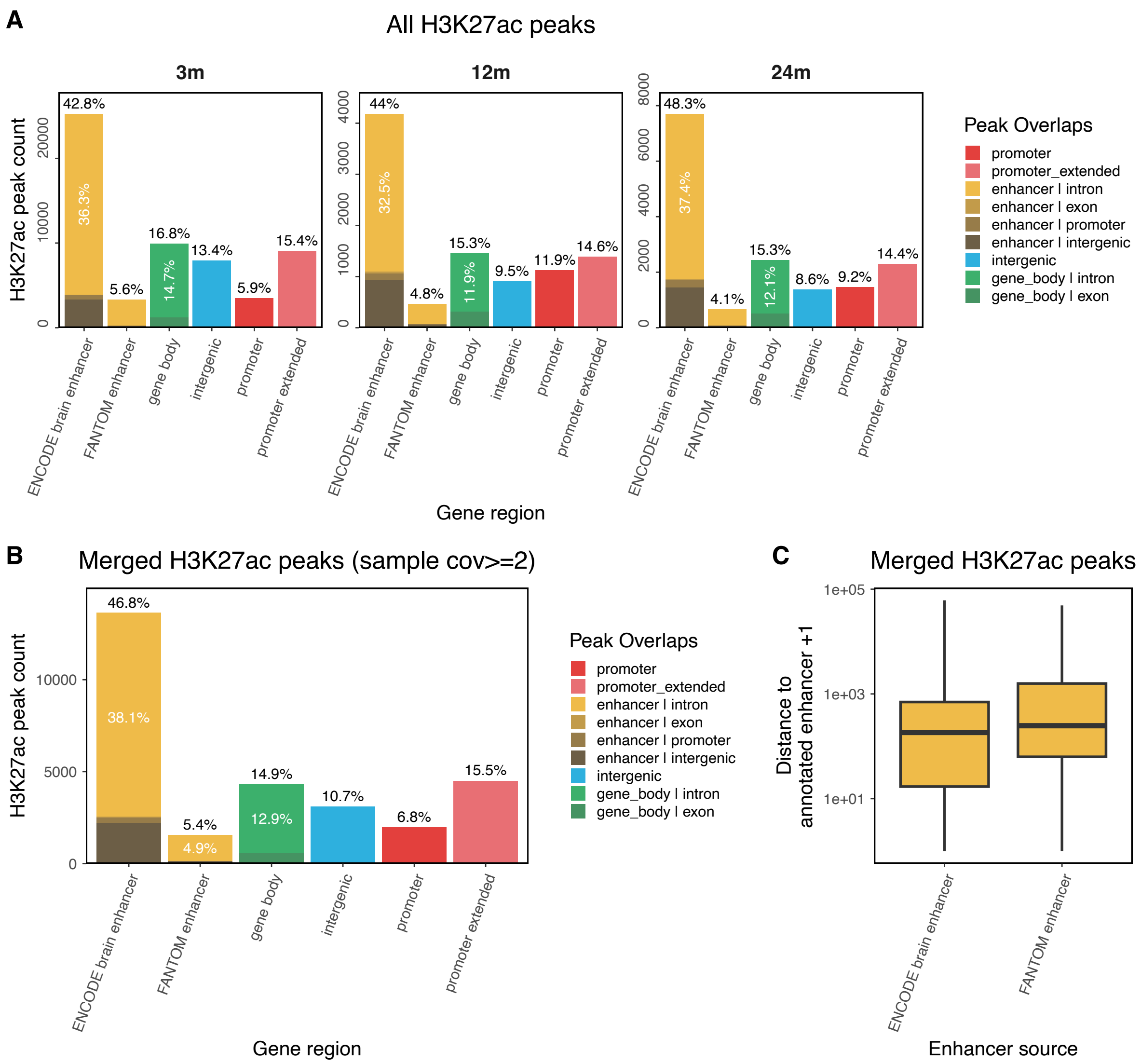

Figure S14. H3K27ac peak locations. (A) Closest annotated features to all H3K27ac peaks by age group. (B) Closest annotated features to the merged set of H3K27ac peaks. Peaks in the merged set were required to be present in at least two samples. (C) Distance to nearest enhancer for all enhancer-adjacent and overlapping peaks. Distance was given a buffer of +1 to enable plotting on a log scale and inclusion of overlapping (distance=0) peaks.

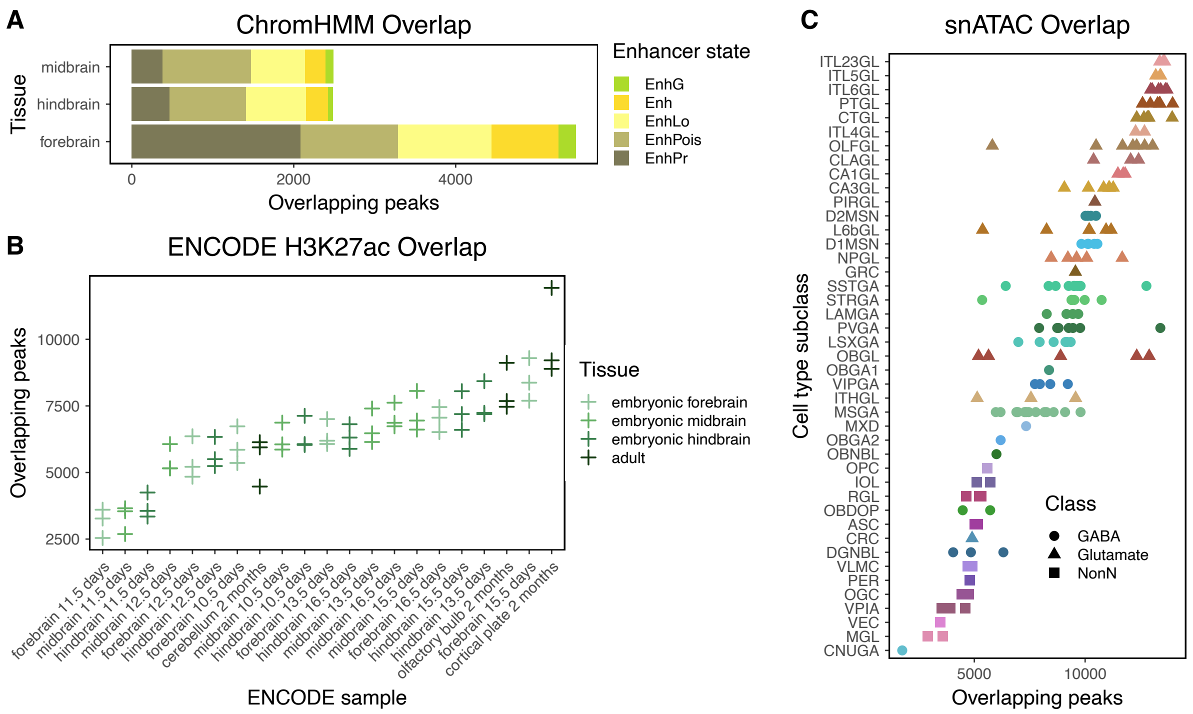

Figure S15. H3K27ac peak overlap with published datasets. (A) Overlap of all replicable H3K27ac peaks with ChromHMM enhancers from ENCODE mouse postnatal midbrain, hindbrain and forebrain. An overlap was considered if the minimum distance between a peak and a ChromHMM region was less than 1000nt. (B) Overlap of all replicable H3K27ac peaks with ENCODE H3K27ac peaks from mouse postnatal brain samples. (C) Overlap of all replicable H3K27ac peaks with snATAC peaks in different cell types from 2-month mouse brains from Li *et al.* (2012). For a table of cell type subclass codes to cell type names and brain locations please see Table S3.

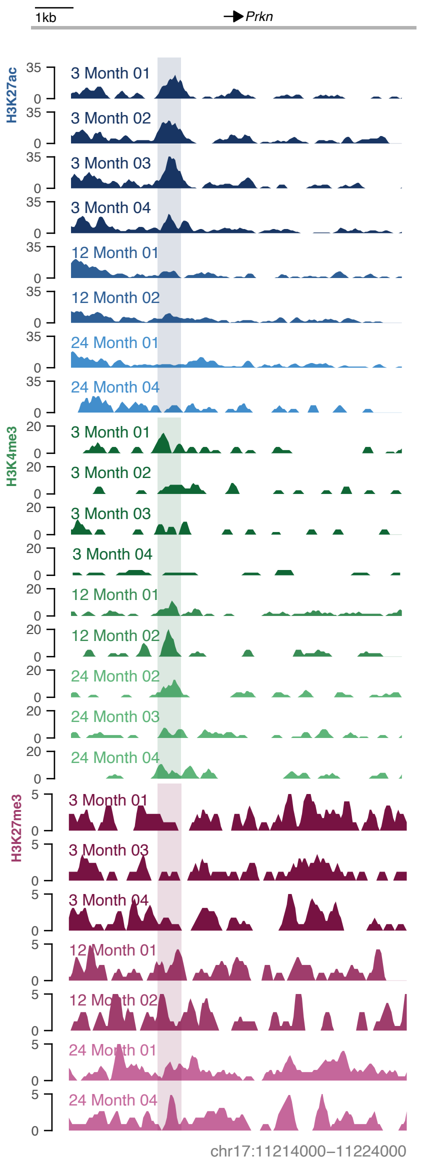

Figure S16. Neuronal H3K27ac, H3K4me3, and H3K27me3 ChIP-Seq signal in a novel H3K27ac peak within an intron of the synaptic signalling gene *Prkn*. Highlighted regions co-occur with H3K4me3 peaks, and do not co-occur with H3K27me3 peaks. Data in this figure relates to Figure 2.

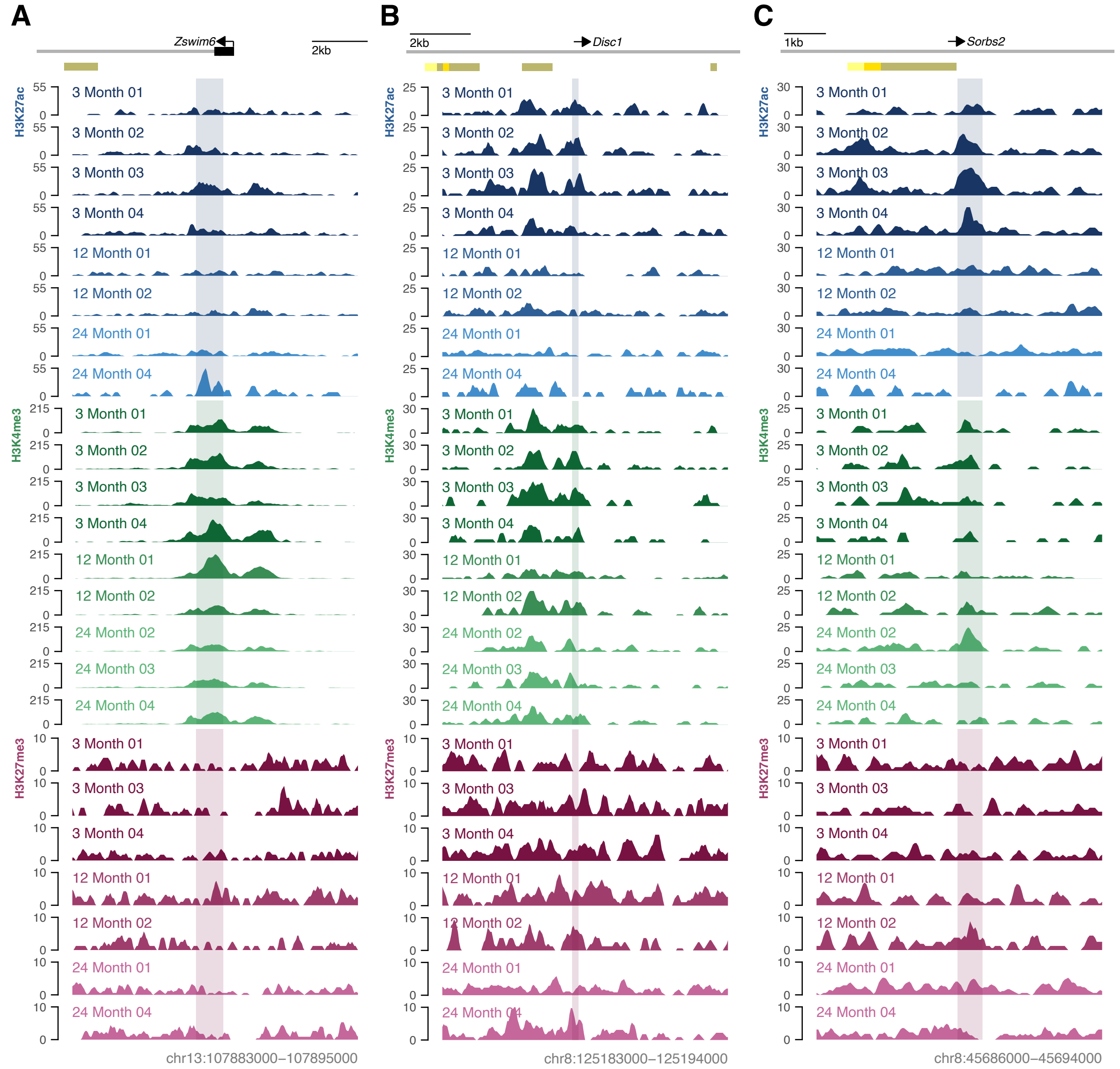

Figure S17. Neuronal H3K27ac, H3K4me3, and H3K27me3 ChIP-Seq signal in representative peaks showing differential H3K27ac modification between 12 and 24 months (A; *Zswim6*), 3 and 12 months (B; *Disc1*) and 3 and 24 months (C; *Sorbs2*). Both *Zswim6* and *Sorbs2* highlighted regions co-occur with H3K4me3 peaks, and no highlighted regions co-occur with H3K27me3 peaks. Data in this figure relates to Figure 3.

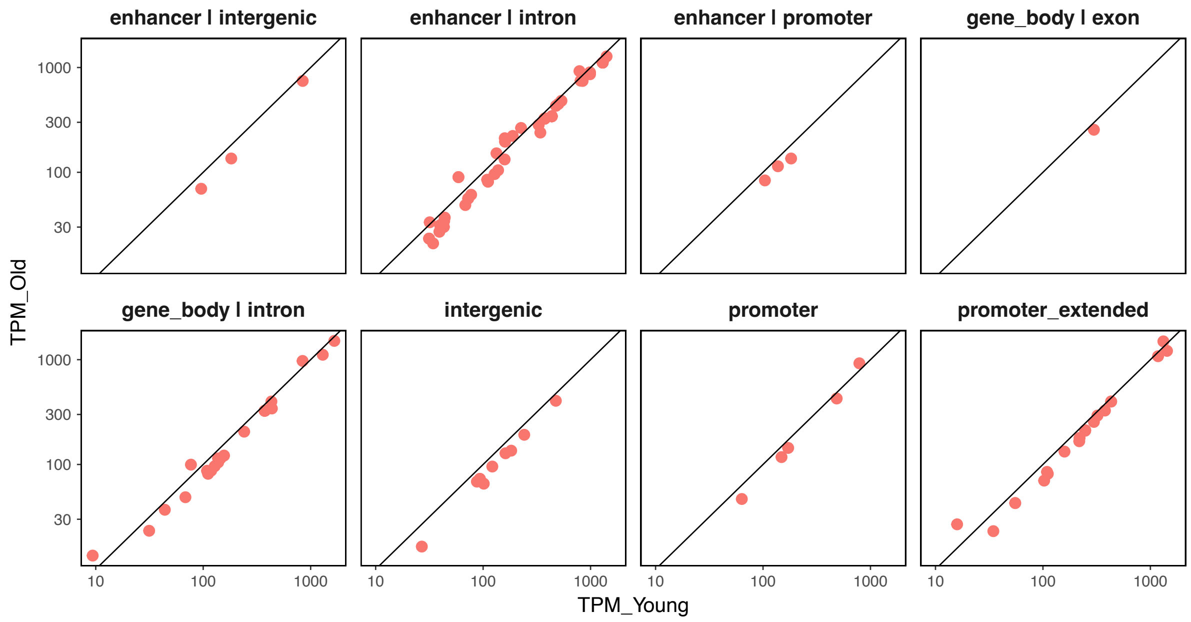

Figure S18. Gene expression changes in genes overlapping or proximal to differentially modified H3K27ac regions between 3 and 12 months. Gene expression values (TPM) were taken from single cell RNA-Seq data in mature neurons (mNEUR) from Ximerakis *et al.* (2019) at both the young and old time points. Only genes which were significantly differentially expressed in mature neurons between young and old age are shown. Each point relates to a distinct genomic region, and a gene with differential expression may be covered by multiple distinct regions of differential H3K27ac modification.

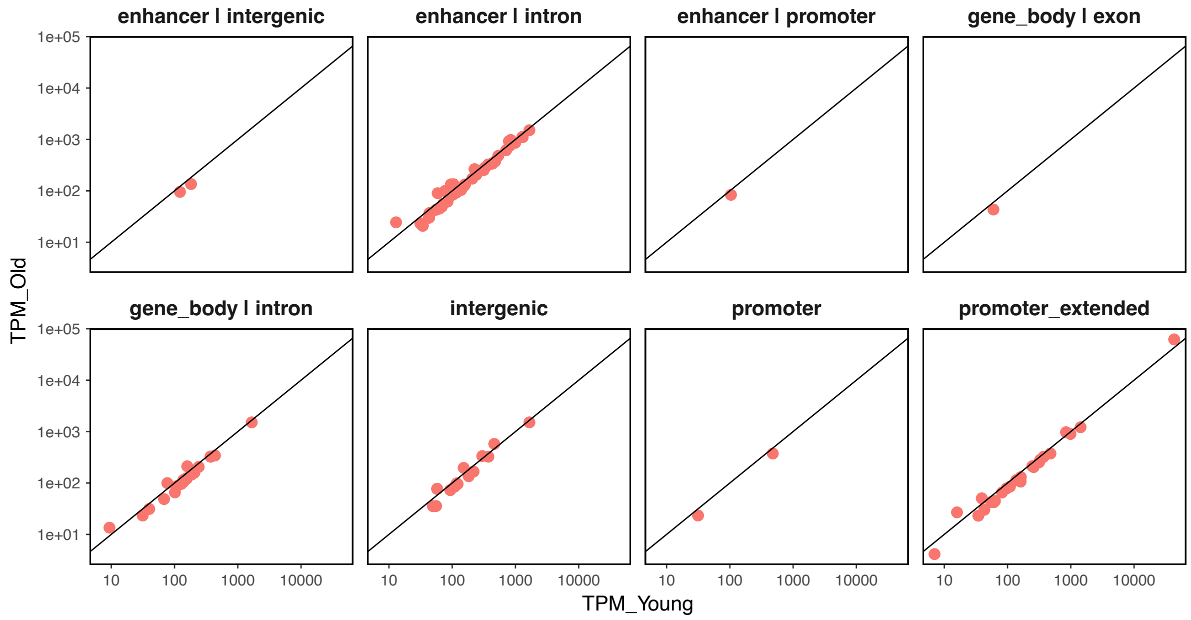

Figure S19. Gene expression changes in genes overlapping or proximal to differentially modified H3K27ac regions between 3 and 24 months. Gene expression values (TPM) were taken from single cell RNA-Seq data in mature neurons (mNEUR) from Ximerakis *et al.* (2019) at both the young and old time points. Only genes which were significantly differentially expressed in mature neurons between young and old age are shown. Each point relates to a distinct genomic region, and a gene with differential expression may be covered by multiple distinct regions of differential H3K27ac modification.

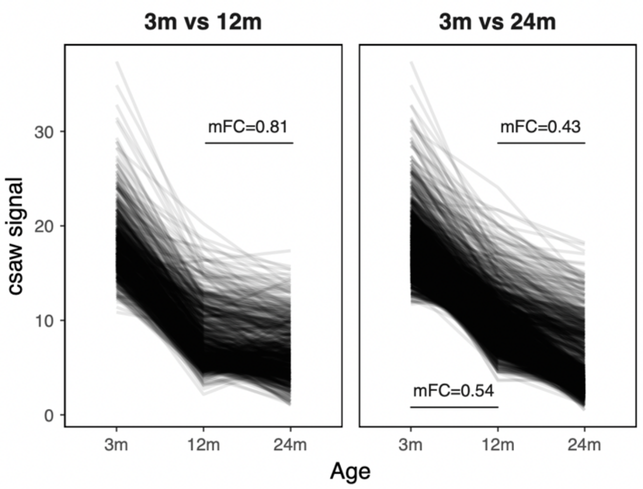

Figure S20. Intermediate ChIP signal changes at regions with significant differential histone modification of H3K27ac between 3 months and 12 months, and between 3 months and 24 months. ChIP signal for each differential region was extracted for each sample and averaged from normalised csaw counts. Median fold change (mFC) for all regions for the additional age comparisons is given above or below the age range.

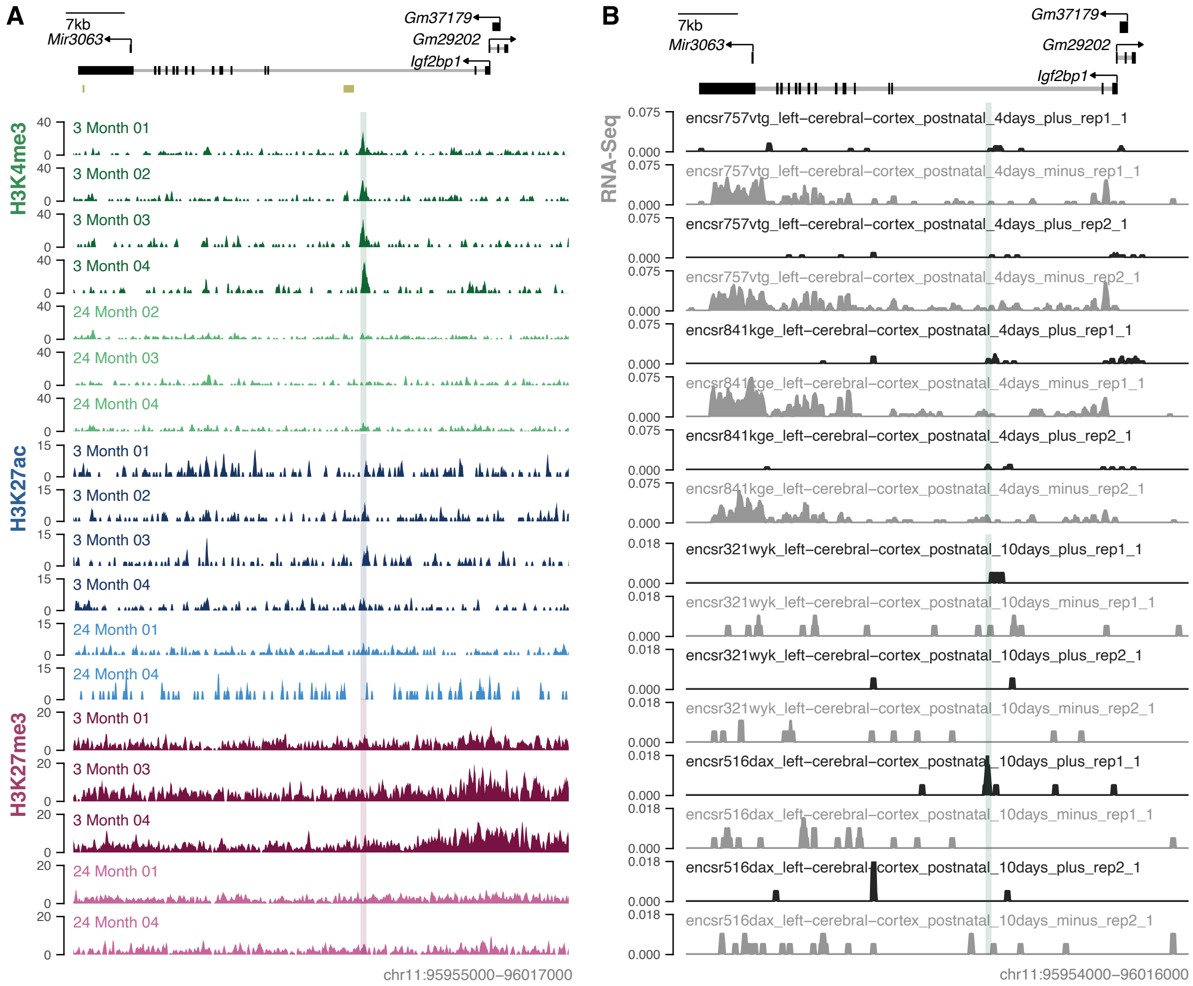

Figure S21. *Igf2bp1* epigenetic profiles and RNA signal. (A) ChIP-Seq profiles for all H3K4me3 (green), H3K27ac (blue) and H3K27me3 (pink) samples. The differentially modified H3K4me3 region is highlighted across all marks. Enhancer track is coloured gold below gene models. (B) RNA-Seq coverage profiles for ENCODE left cerebral cortex samples at postnatal days 4 and 10. Signal from the plus strand (i.e. opposite strand to *Igf2bp1*) is coloured black, and signal from the minus strand is coloured grey. Differentially bound H3K4me3 region is highlighted green across all samples.

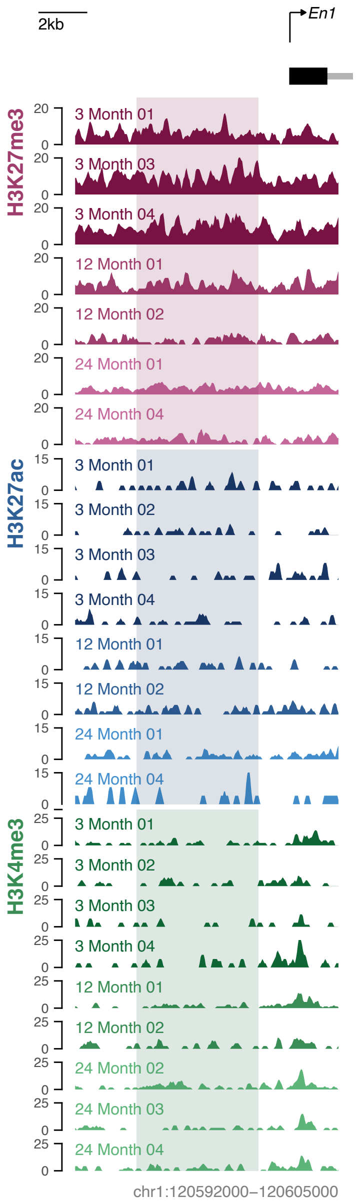

Figure S22. Neuronal H3K27me3, H3K27ac, and H3K4me3 ChIP-Seq signal in representative peaks showing differential H3K27me3 modification between 3 and 24 months (*En1*). Data in this figure relates to Figure 5C.

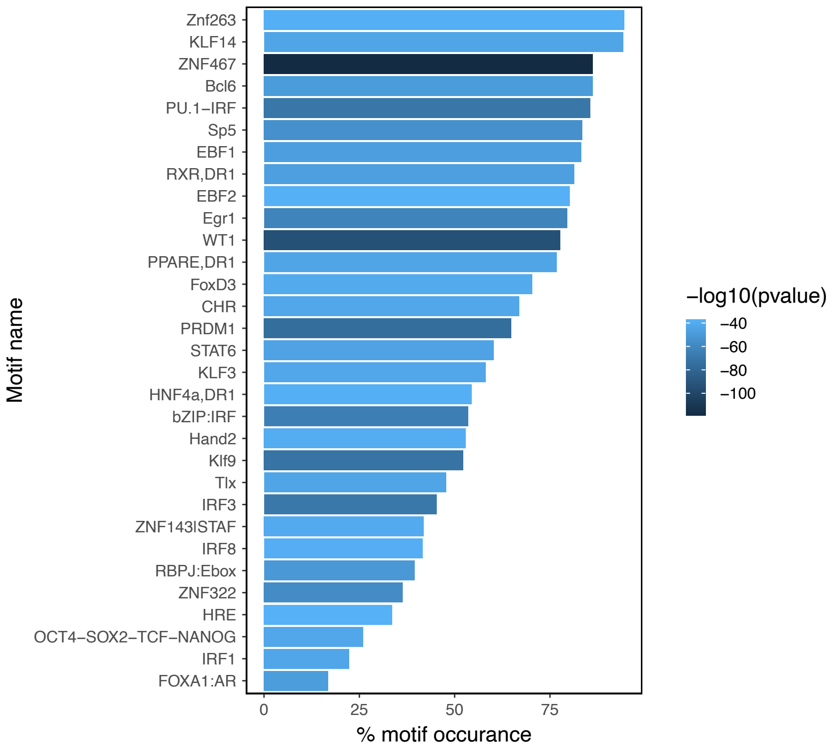

Figure S23. Motif enrichment of promoter regions of H3K27me3 peaks lost with age. Promoter-overlapping peaks were defined as those overlapping the region 5kb upstream to 1kb downstream from the TSS, as in Figure 5A.

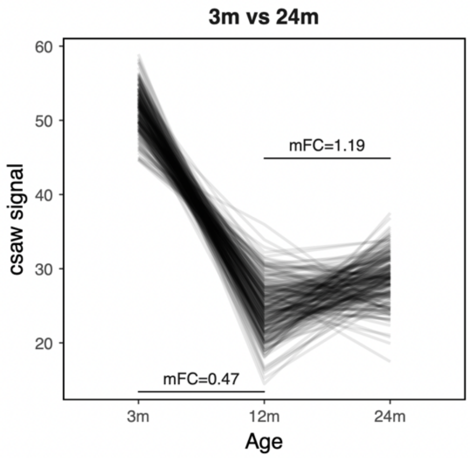

Figure S24. Intermediate ChIP signal changes at regions with significant differential histone modification of H3K27me3 between 3 months and 24 months. ChIP signal for each differential peak was extracted for each sample and averaged from normalised csaw counts. Median fold change (mFC) for all regions for the additional age comparisons is given above or below the age range.

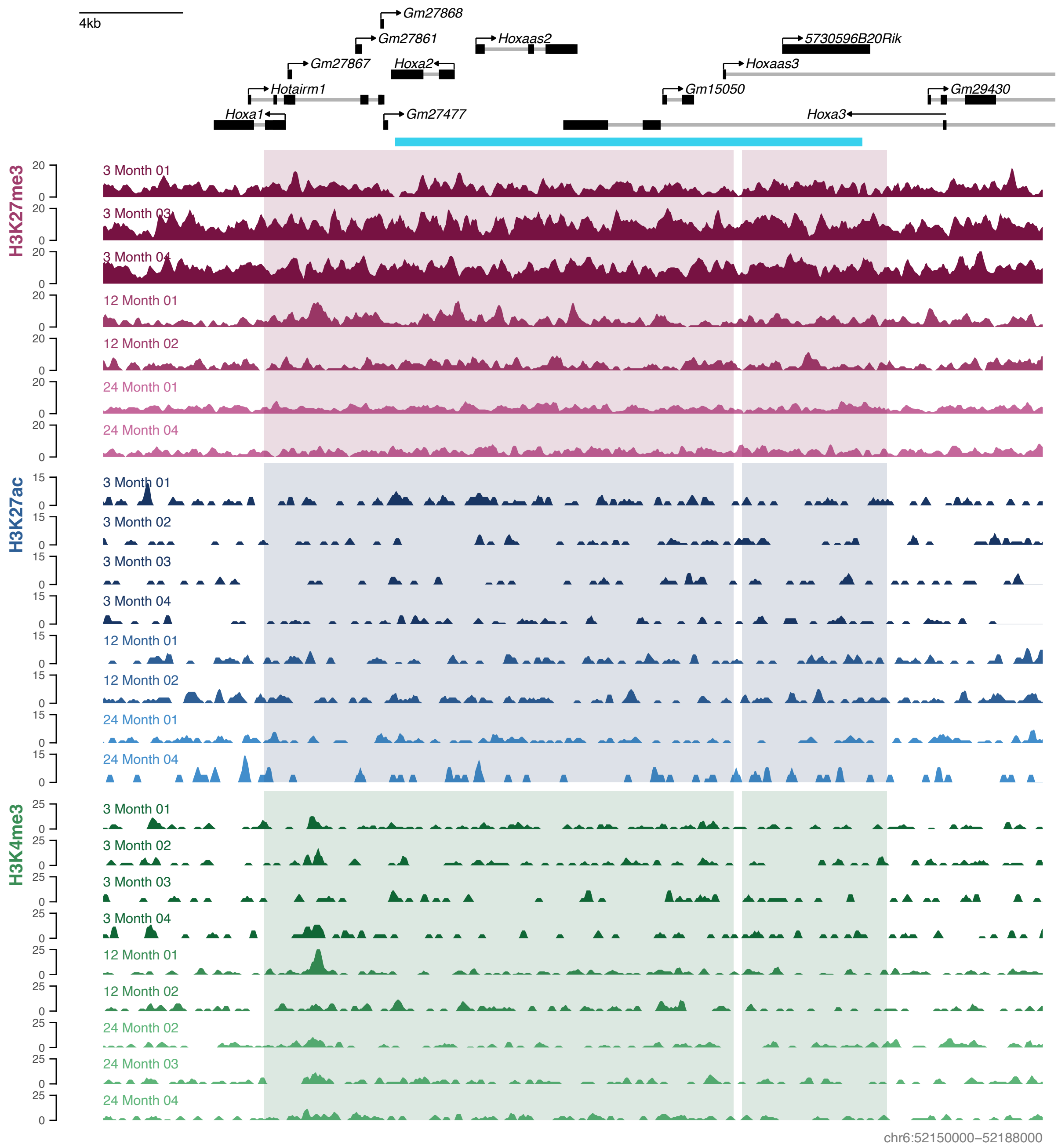

Figure S25. Neuronal H3K27me3, H3K27ac, and H3K4me3 ChIP-Seq signal in the E14.5 Brain mouse super enhancer region (light blue; top). Data in this figure relates to Figure 5E.

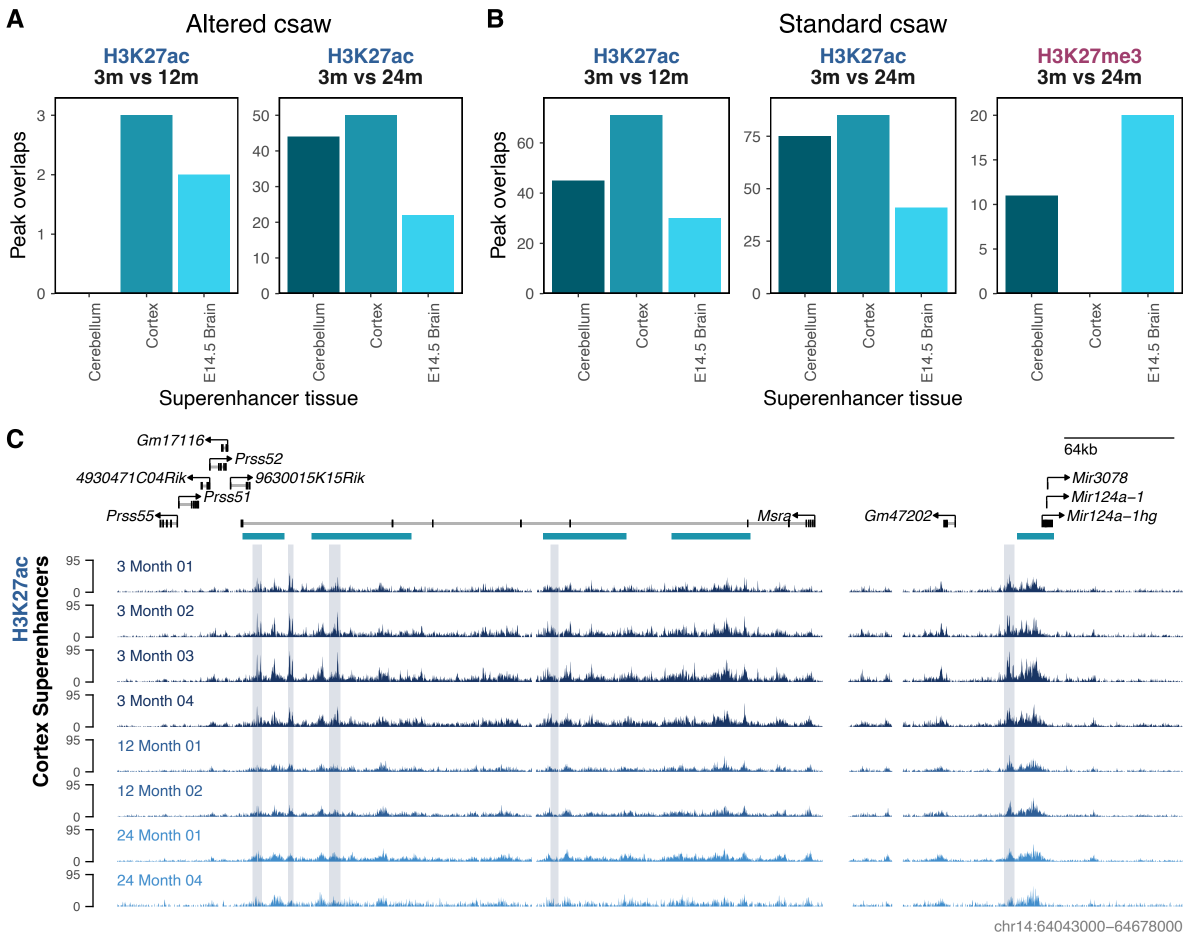

Figure S26. Cooccurrence of differentially modified regions and annotated brain superenhancer regions. (A) Overlap between differential regions from the altered (TMM normalised) csaw analysis and Superenhancers from the mouse cerebellum, cortex, and E14.5 brain. (B) Overlap between differential regions from the standard (non-linear normalised) csaw analysis and Superenhancers from the mouse cerebellum, cortex, and E14.5 brain. (C) Representative region of differential H3K27ac (highlighted regions) loss in cortex superenhancer regions (teal track).

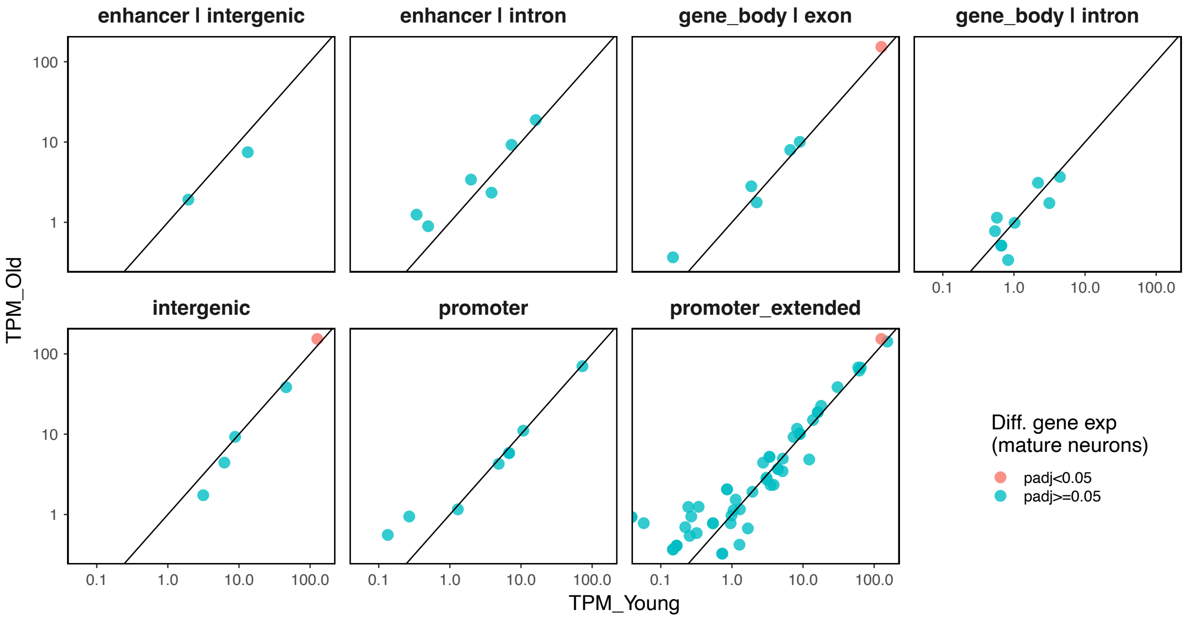

Figure S27. Gene expression changes in genes overlapping or proximal to H3K27me3 regions differentially occupied between 3 and 24 months. Gene expression values (TPM) were taken from single cell RNA-Seq data in mature neurons (mNEUR) from Ximerakis *et al.* (2019) at both the young and old time points. Both genes which were significantly differentially expressed (red) and showed no significant change (blue) in mature neurons between young and old age are shown. Each point relates to a distinct genomic region, and a gene with differential expression may be covered by multiple distinct regions of differential H3K27me3 modification.
